# From a weighing scale to a pole: a comparison of two different dosage strategies in mass treatment of *Schistosomiasis haematobium*

**DOI:** 10.3402/gha.v7.25351

**Published:** 2014-12-03

**Authors:** Per Nordin, Gabriele Poggensee, Sabina Mtweve, Ingela Krantz

**Affiliations:** 1 The Skaraborg Institute for Research and Development, Skövde, Sweden; 2 Nigerian Field Epidemiology and Laboratory Training Program, Abuja, Nigeria; 3 Department of Community Health, Kilimanjaro Christian Medical Centre, Moshi, Tanzania

**Keywords:** praziquantel, dosage, mass treatment, S. haematobium, dose-pole, underdosage, overdosage

## Abstract

**Background:**

Clinical schistosomiasis in endemic countries is treated with a single dose of praziquantel per 40 mg/kg body weight. Treating according to weight, in resource-poor settings when thousands of doses are to be administered in mass treatment campaigns, is considered problematic. A calibrated dose-pole based on height was developed and is now used in mass treatment campaigns for determining the doses for schoolchildren. The dose-pole will generate dose errors since every child population contains individuals that are either short or tall for weight. The aim of this study is to explore whether the WHO praziquantel pole is a satisfactory dose instrument for mass treatment of *S. haematobium*.

**Methods:**

In 1996 and 2002, 1,694 children were surveyed in the Kilimanjaro Region, Tanzania. We compared doses given by weight to doses given by height using descriptive statistics and regression.

**Conclusions and interpretation:**

The WHO dose-pole for praziquantel is based on height of the patient; however, children with the same height will differ in weight. Our study shows that children with the same weight could qualify for up to four different dose levels based on their height. The largest variation of doses based on the WHO dose-pole will be found in children below 20 kg of bodyweight. Using bodyweight and tablet halves as the smallest tablet division unit to determine the doses of praziquantel, one only has to identify every 6th kilogram of bodyweight; the doses will then vary a lot less than when using the WHO dose-pole.

Schistosomiasis is a neglected tropical disease with at least 200 million infected individuals. It causes damage to internal organs which could lead to serious sequelae in the urinary and gastrointestinal tracts. An estimated 650 million people live in endemic areas (1).

According to WHO recommendations, clinical schistosomiasis in endemic countries should be treated with a single dose of praziquantel per 40 mg/kg body weight (1). This single dose is also recommended in mass treatment campaigns of schoolchildren and adults considered to be at risk. To treat according to body weight in resource-poor settings when thousands of doses are to be administered is considered problematic due to a substantial risk of technical and systematic measurement errors (2). A poor scale would generate severe errors in dosing (3, 4).

A calibrated pole based on height was suggested and developed as an alternative for determining the dose for schoolchildren (1, 4) and is now used in mass treatment programs (5–7). Originally, the dose-pole was designed for children between 110 and 178 cm. Later on, children between 94 and 109 cm as well as those above 178 cm were included (2). The WHO mass treatment recommendations also include selected adults at risk in highly and moderately endemic areas (1). In practice, WHO recommendations mean a dose of the divisible 600 mg praziquantel tablet is to be given per body height category (Table 1).

**Table 1:** Doses of praziquantel in numbers of 600 mg tablets by height in cm according to the WHO dose-pole

Height (cm)	Number of tablets
94–109	1
110–124	1.5
125–137	2
138–149	2.5
150–159	3
160–178	4
>178	5

Modified after WHO (1).

By definition, this approach will always have in-built errors that could lead to both over- and underestimation of the praziquantel dose; every child population will contain individuals that are either short for weight or tall for weight. The distribution of body weight for each given height will vary depending on what type of child population is under consideration. Consequently, in every population, there will be some individuals who will get a dose outside the recommendations, even if a dosage interval, allowing a lower and upper limit of 30–60 mg/kg respectively, is introduced (8). This calibration problem has customarily been handled by adjustments of the intervals based on height, instead of weight, to maximize the number of individuals getting a dose considered as acceptable with regards to both toxicity and efficacy (4). Underdosing has been brought forward as an important risk factor for the development of resistance (9). Development of resistance and side effects of praziquantel in mass treatment programs of *S. haematobium* are, however, not extensively studied (10).

The aim of this study is to explore in both theory and practice whether the WHO praziquantel pole is a satisfactory dose instrument for mass treatment of *S. haematobium*, or whether some other approach could be more appropriate in order to avoid under- and overdosage.

## Subjects and methods

### Subjects

The project ‘Sustainable Prevention of Endemic Schistosomiasis’ (SPES) has surveyed schoolchildren, mainly in standard/class four, from the Kileo and Kivulini villages of the Kilimanjaro Region in Tanzania since 1996 (11). For each examined child, the age, sex, weight, height and *S. haematobium* egg occurrence were recorded. Weight was measured once by a spring scale, height once by a measuring tape affixed to the wall. These measurements were conducted by the same trained public health nurses throughout the years. Altogether, 1,694 children were surveyed in 1996 or 2002 – 837 (49%) girls and 857 (51%) boys with age ranging from 5 to 18 years. Complete observations regarding age, sex, weight and height were obtained from 1,477 (87%) of these children – 736 (50%) girls and 741 (50%) boys (Table 2), which constitutes the empirical material. The theoretical deliberations are based on the manufacturers’ dosage recommendation, 40 mg/kg.

**Table 2:** Mean values, standard deviations, coefficients of variation, minimum and maximum values for the variables age, weight and height among the 1,477 schoolchildren

Variable	Mean	Sd	CV	Min.	Max.
Age in years	11.0	2.82	25.6	5	18
Weight in kg	27.3	8.02	29.4	14	65
Height in cm	130.4	12.88	9.9	99	170

### Statistical methods and data analysis

We compared doses given by weight to doses given by height with descriptive statistics [proportions, means standard deviations (Sd), coefficient of variation (CV), range] and linear regression. We examined the differences between the two approaches using a Bland-Altman analysis for equality of measures (12). Ninety-five percent confidence limits are given in square brackets. The level of significance is set at 5%.

### Ethical considerations

The surveys were approved by the Kilimanjaro Christian Medical Centre/Kilimanjaro Christian Medical University College Ethical Committee and the Regional Medical Officer, Kilimanjaro Region. The school authorities gave their consent after having been informed of the purpose of the surveys. All schoolchildren were invited to participate after informed consent from their parents or guardians had been obtained. All children affected with schistosome infections were treated free of charge with praziquantel [single oral dose of 40 mg/kg b.w. as recommended by WHO (1)].

## Results

The dose-pole is based on the idea that height can be used instead of weight to determine the dose (4), consequently the extent of variation found in weight should also be found in height. However, these two variables are measured in different units (kg and cm); thus the Sds are not directly comparable. The CV – a statistical measure not bound to any units of measurement – shows that weight varies to a larger extent than height in our study. The observed CV for weight is notably three times larger than the CV for height (Table 2).

### The association between weight and height

**Fig. 1:**
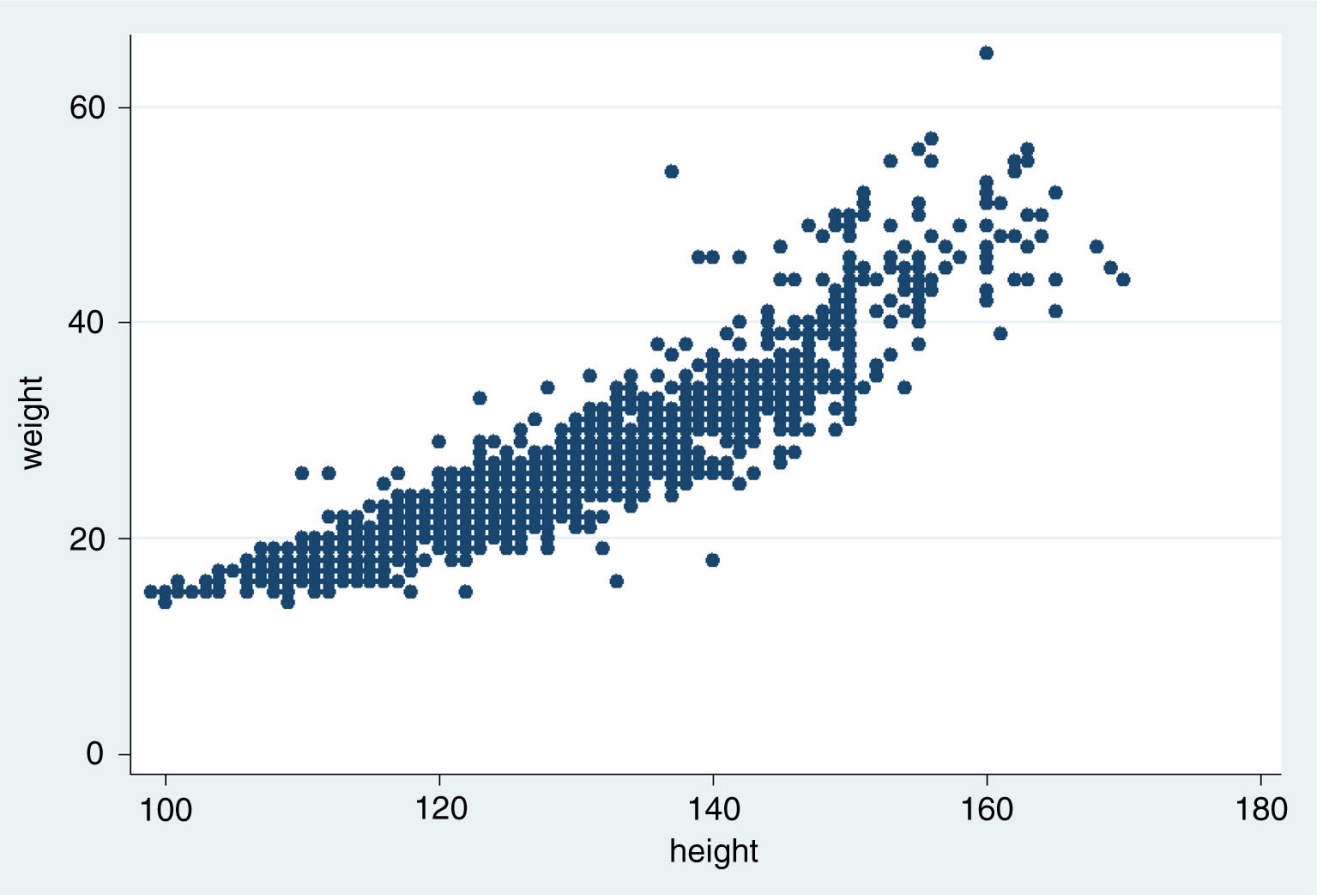
A scatter plot of height in cm and weight in kg for 1,477 schoolchildren with a correlation coefficient (r) of 0.9.

In Fig. 1, the scatter plot shows a slightly J-shaped relationship between weight and height. The variation increases as weight and height increase; many values of height are found for each value of weight.

Nevertheless, the variation in height explains the variation in weight to quite a large extent as expressed in the coefficient of determination (*r* ^2^=0.82). What we see is a good correlation, but not real evidence for applicability when translated to the administration of praziquantel. When examining agreement of measures, the correlation coefficient is not the only issue to consider (12).

### Consequences of the recommendation from the drug manufacturers

When using weight, as recommended by the praziquantel drug manufacturers, deviations in dosages are difficult to avoid, since the tablet is produced as a divisible 600 mg unit. The commonly manufactured praziquantel tablet (Biltricide^®^) has three parallel scores, which means it can be broken into four segments. To divide the tablet into smaller segments than halves would result in a more precise dose, but is a procedure probably not feasible during mass treatment campaigns (13).

**Fig. 2:**
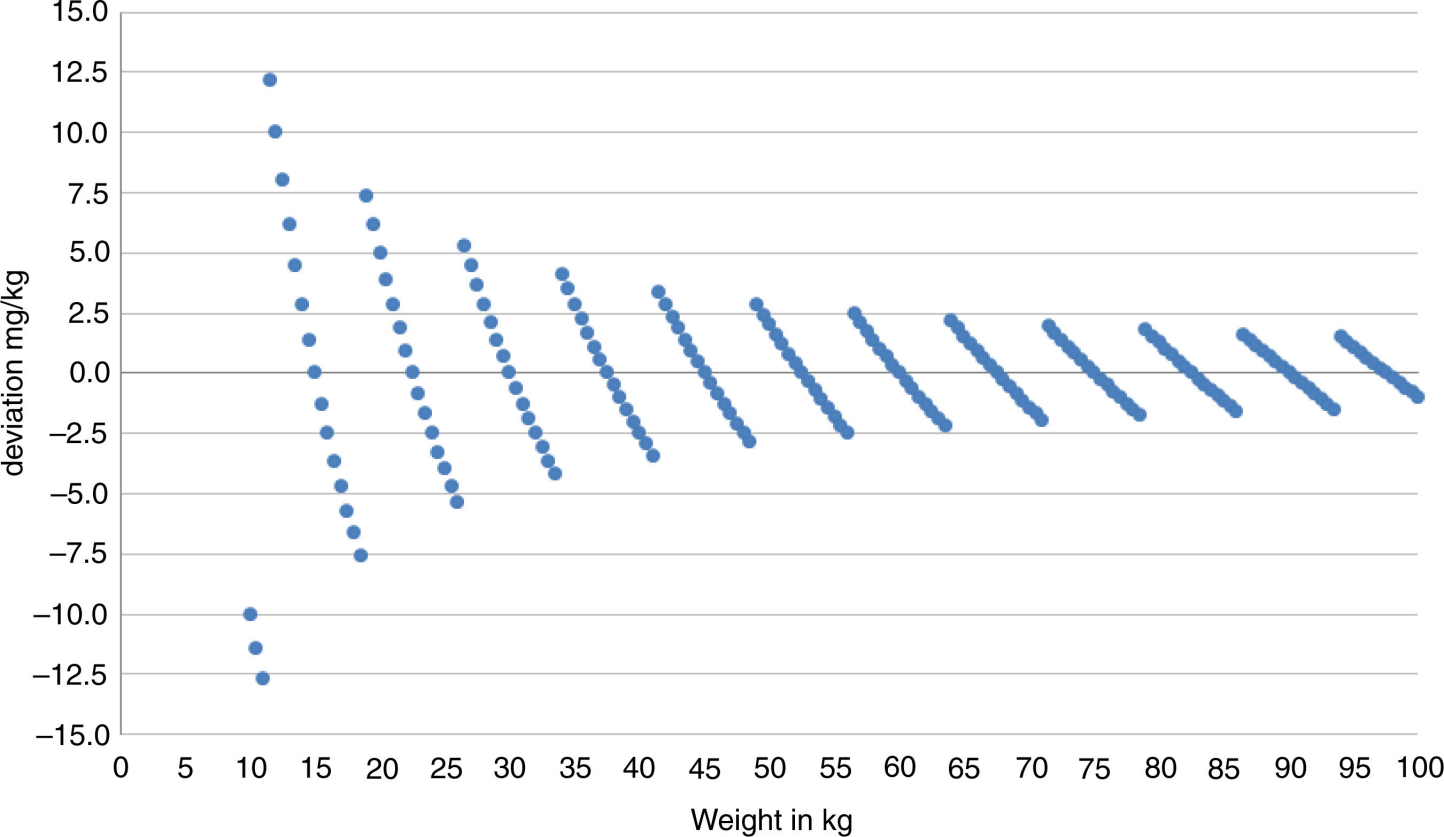
Theoretical deviations in mg/kg according to body weight, illustrated by trajectories, when 40 mg/kg is the target dose and 300 mg tablet halves are used as the smallest dose unit.

Given that weight could be measured accurately and only tablet halves used as recommended by WHO (1), the maximum deviation in dose could never be more than 300 mg per individual. This may be of little importance for a child of moderate to heavy weight or an adult. Theoretically, however, for a child of low weight, this would mean a departure from the recommendation of 40 mg/kg as illustrated by the trajectories in Fig. 2. On the other hand, the children in our study vary in weight from 14 to 65 kg (Table 1), which means that the actual dose could deviate from the recommended dose by as much as ±7.5 mg/kg for those with the lowest weight to ±2.5 mg/kg for those with the highest weight.

From Fig. 2, it can also be deduced that the weight approach, which is based on division of tablets, will result in a globally valid categorization of individuals into determined weight classes. Furthermore, using tablet halves will generate a weight versus dose relationship as illustrated by the range of weight of the children in this study (Table 3). We thereby arrive at eight levels of the dose, from 600 to 2,700 mg in 300 mg steps.

**Table 3:** Praziquantel doses according to weight applied to the weight range of 1,477 schoolchildren when tablet halves are used as the minimum segments

Weight (kg)	Number of tablets
12–18	1
19–26	1.5
27–33	2
34–41	2.5
42–48	3
49–56	3.5
57–63	4
64–71	4.5

### Comparison between the WHO dose-pole and the manufacturers’ recommendations

**Fig. 3:**
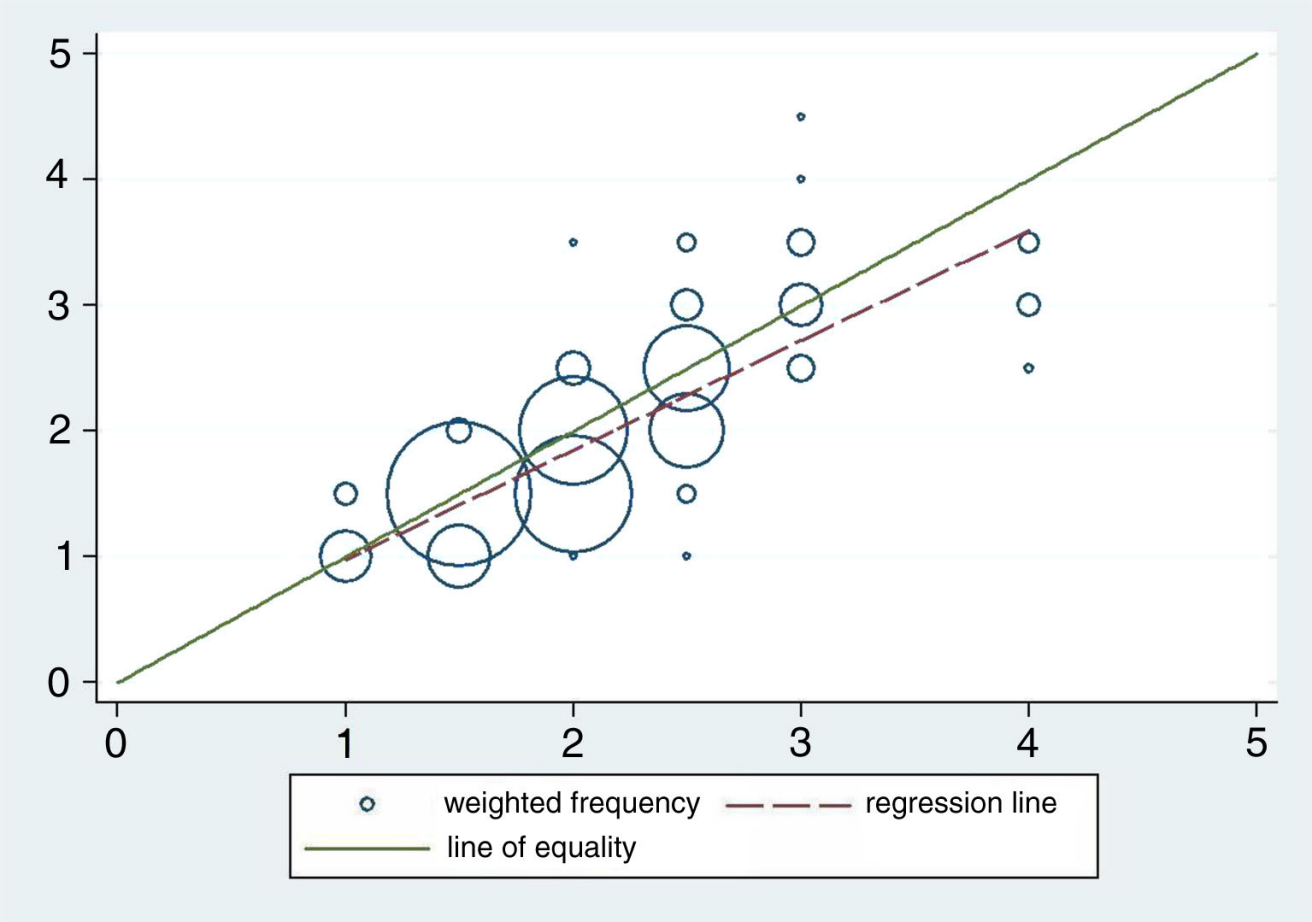
Frequency weighted scatter plot with doses in number of pills from the dose-pole on the horizontal axis and number of pills by weight on the vertical axis with both the line of equality and the fitted line included.

How do these two approaches (dose-pole and weight) of administering praziquantel relate to one another given that the relationship in Table 3 is regarded as the ‘gold standard’? The choice of the ‘gold standard’ is motivated by the fact that it conforms more closely to the manufacturers’ recommendations. The scatter plot in Fig. 3 depicts how these two ways of deciding the dose correspond to each other. The data points are presented as circles where the size of the circle represents the number of observations in each point. If the methods were in perfect agreement, the circles in the plot would be centered on the line of equality (y=x). In this study, deviations from the line of equality are evident. Furthermore, there is a systematic difference between the two methods illustrated by the fact that the fitted line has a slope slightly different from the line of equality (*b*=0.87 and *p*<0.001).

**Fig. 4:**
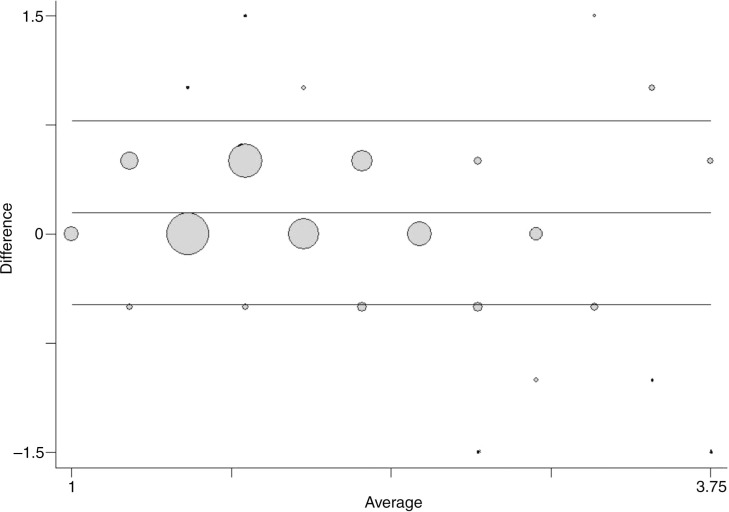
Frequency weighted scatter plot of the differences in doses on the y-axis against the mean values of doses on the x-axis when comparing doses given by the dose-pole and doses using the weight approach. Reference lines included for mean differences as well as the limits of agreement.

Figure 4 shows a Bland-Altman plot for comparison of measures applied using our data. Again, the data points are presented as circles, where the size of the circle represents the number of observations in each point. The mean difference is 0.14 [0.13; 0.16], showing that a systematic difference exists and that the use of the dose-pole would *on the average* mean a slightly but statistically significant higher dose than the ‘gold standard’.

When comparing the two methods, a small proportional error, that is, a linear trend in the differences, is seen along the mean (*b*=−0.052 and *p*=0.001). There is also a slight error which is dependent on the size of the measurement. The variance increases slightly with the average size of the dose (Fig. 4).

Although the underlying assumptions for using and interpreting the limits of agreement (12) have not been completely met, we do not consider the departures from these assumptions large enough to invalidate our results. The limits of agreement (=the reference range for differences) are −0.49 to 0.77, meaning that approximately 95% of the differences are within these limits. In other words, circa 95% of the children would have received a dose using the dose-pole, which would range from ½ a tablet less to ¾ of a tablet more than the weight approach. The 95% confidence intervals for the limits of agreements are relatively narrow [−0.54; −0.43] and [0.72; 0.83], respectively. However, it is important to acknowledge that there are difficulties in assessing whether these limits of agreement are too wide unless considering the dose in relation to the weight of the subjects.

When comparing the doses given by the dose-pole to doses given by weight using tablet halves – but now considering the dose in mg/kg – it is obvious that the dose-pole approach is less accurate than the one based on weight (Table 4). This result is not a direct consequence of our earlier observation of the difference in the CV between weight and height. It is true that height does not fully explain the variation seen in weight; thus, a dosage method based on height will not reach the same precision as the method based on weight. The point here is that in practice *both* methods rely on a categorization of a continuous variable. In the case of the dose-pole, this is clear from the definition. However, as noted in Table 3, the weight approach relies on a categorization of weight. Aiming at a dose of 40 mg/kg, most of the doses given by the dose-pole lie in an interval from −10 to +20 mg/kg around the target dose (Table 4). The distribution is skewed with a clear tendency to doses larger than the target, yet there are cases when the dose-pole misclassifies children to almost 20 mg/kg too low a dose. At the other end of the distribution, the pole provides doses close to double the recommended dose. The largest deviations from the target dose appear as overestimations of the dose and are found in children with relatively low weight. On the average, the dose-pole generates doses slightly higher than required for our child population with a mean of 43 mg/kg. Furthermore, the dose-pole gives doses with a higher variation compared to the approach with weight and tablet halves (Sd_pole_=6.0, Sd_weight_=3.6).

**Table 4:** Deviations in mg/kg from the target dose of 40 mg/kg, and number and percentage of children using the WHO dose-pole and the weight approach with tablet halves

		Number and proportion of children with different extent of deviation from target dose when			
		
Dose classification	Deviation from target (mg/kg)	Using the dose-pole	%	Using weight	%
	41–45	1	0.1		
Higher than	36–40				
target	31–35	1	0.1		
	26–30				
	21–25	4	0.3		
	16–20	44	3.0		
	11–15	89	6.0		
	6–10	406	27.5	63	4.3
	1–5	508	34.4	629	42.6
On target	0	53	3.6	89	6.0
	1–5	301	20.4	648	43.9
Lower than	6–10	66	4.5	48	3.2
target	11–15	2	0.1		
	16–20	2	0.1		

A little more than 25% of the measured children would receive a dose below target with the dose-pole as compared to 47% with the weight approach. The relation is reversed for doses higher than target, 71 and 47%, respectively. The more extreme deviations, however, both at the higher and lower ends, are all produced by the dose-pole; more than 9% of the doses were 11 mg/kg or more above the target dose. Some cases with doses lower than target by 10 mg/kg or more were also noted. The mean dose clearly differs, but even more so, the extent of variation differs between the two methods (Table 4).

**Fig. 5:**
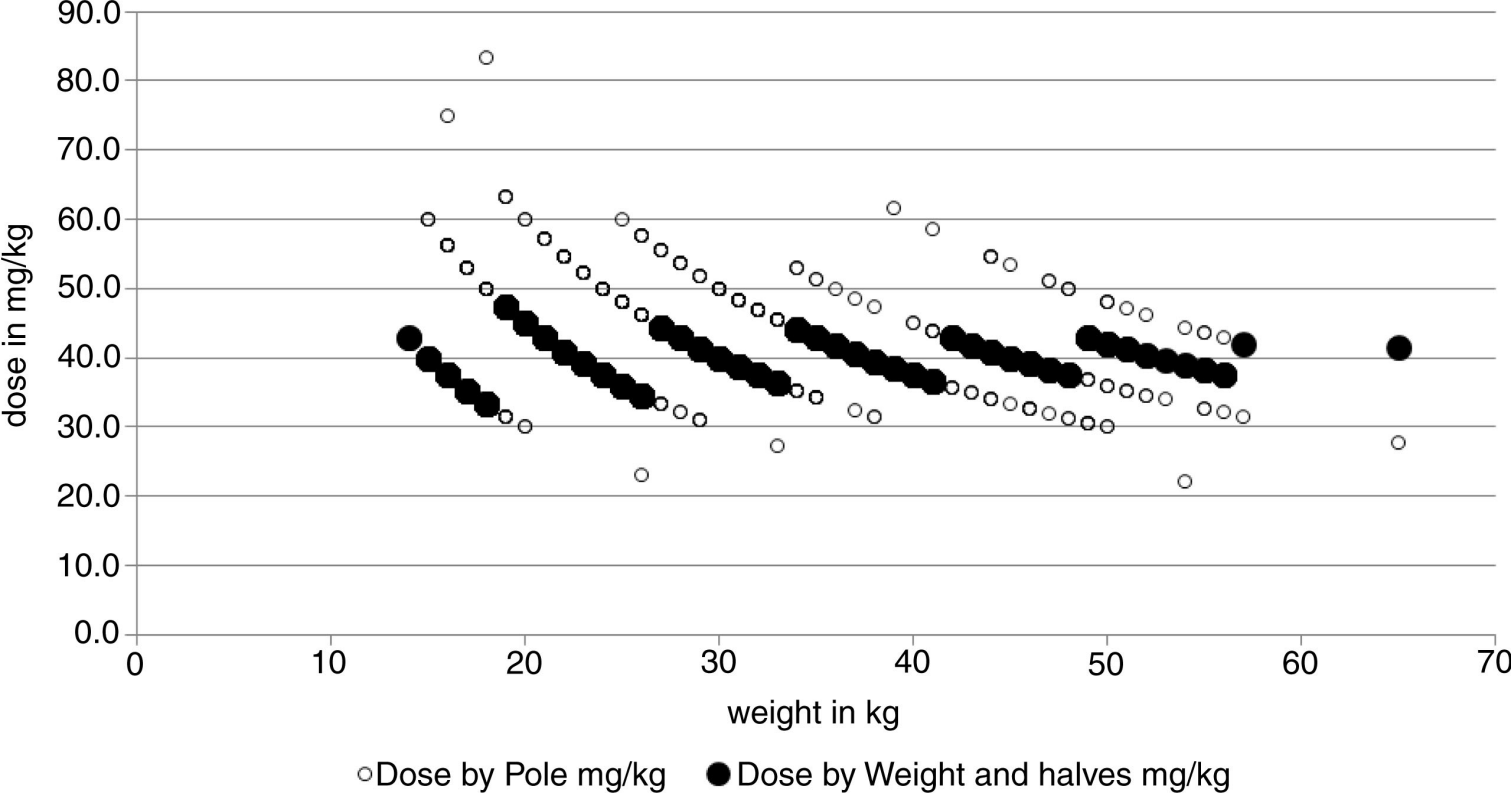
Dose by the dose-pole and dose by weight and tablet halves in mg/kg, all according to bodyweight.

**Fig. 6:**
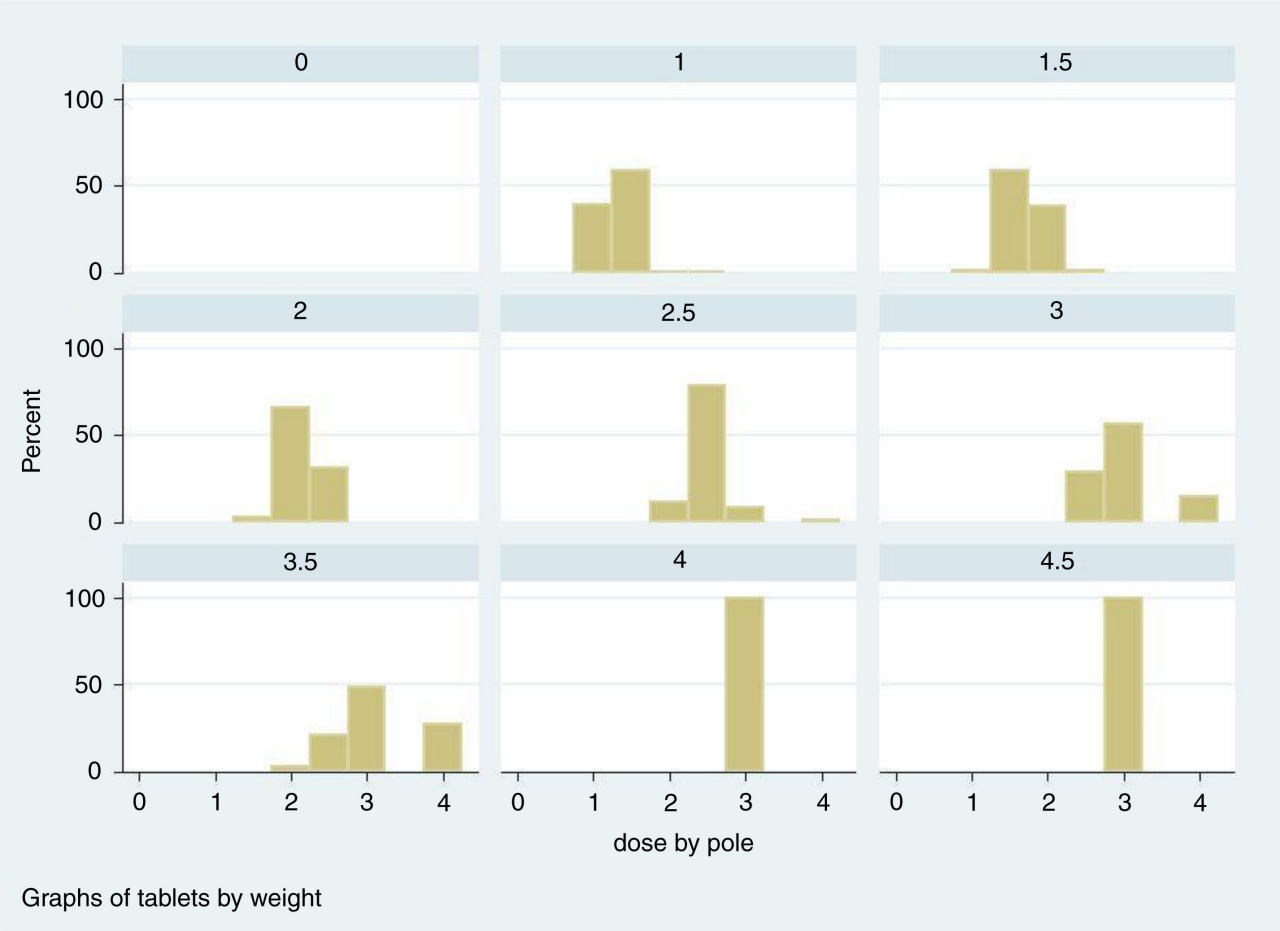
Histograms of doses given by the dose-pole for each dose given by the weight and halves approach.

A detailed illustration of the problem is given in Fig. 5, where the dose outcomes by the two methods are plotted according to the weight of the children in our study. Essentially, this gives the same message as Table 4, but here we also see the trajectories as in Fig. 2. The trajectories represent the different numbers of tablets according to both methods. The filled circles stand for the weight and tablet halves approach. These trajectories are non-overlapping for weight. The hollow circles represent the dose-pole approach; different children with the same weight will receive different doses simply because their heights differ. In our child population, it would mean that children with the same weight qualify for three different dose levels and sometimes even four (Fig. 6). The dose-pole also appears to give the largest deviations from the target dose in children below 20 kg (Fig. 5).

From Fig. 6, it is evident that a child could be given as much as 1½ tablets too many or too few in comparison to the ‘gold standard’. Translated into number of children, this means that 31 of the 1,477 children in our population would have received either one or more tablets too many (21) or one or more tablets too few (10).

**Fig. 7:**
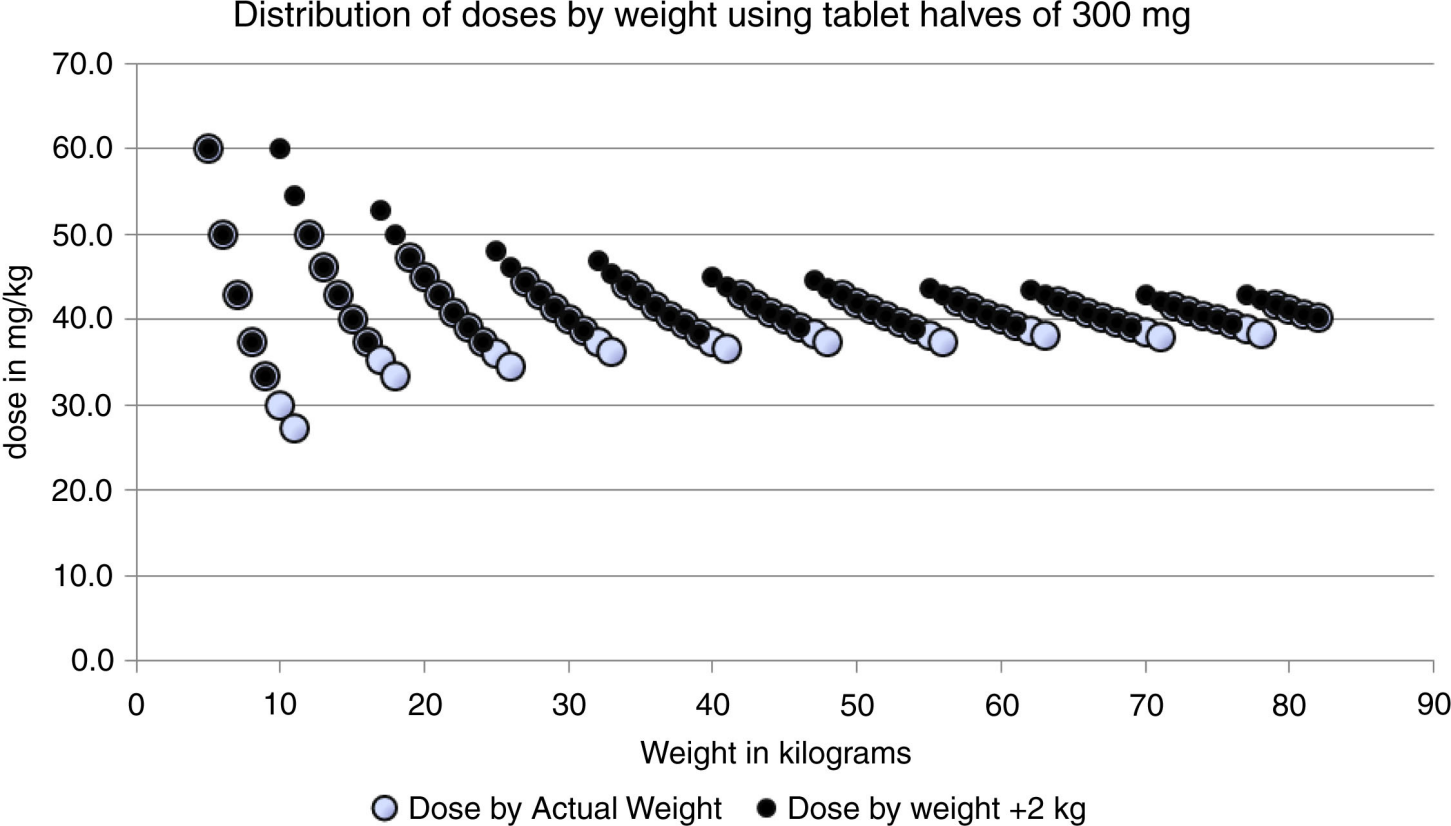
Theoretical distribution of doses (mg/kg) in the case of an accurate scale and when the scale overestimates the weight by 2 kg.

To illustrate that the scale-based method will provide doses with less inherent variation, we show two different scenarios in Fig. 7. Two measurement series are presented, one based on an accurate scale and one based on a scale which produces an overestimate of the weight by 2 kg. The hollow circles show the distribution of doses with an accurate scale using an arbitrary weight range from 5 to 83 kg. As shown before, the distribution of doses is not overlapping and is accurate as well, except for children with a weight below 15 kg. Below this weight the inherent variation starts to show. There is only one situation where the resulting dose goes slightly below 30 mg/kg, namely, at 12 kg body weight. The reason for this is not due to the scale, of course, but is a simple consequence of the fact that we are assuming tablet halves are the smallest practically possible division. If there was a way to easily and repeatedly divide this tablet into quarters or use a syrup preparation, this problem would cease to exist.

The filled circles represent the situation where the scale consistently overestimates the weight of the person by 2 kg; the effect on the dosage is indeed very moderate. A few cases will now get a slightly higher dose. The lowest dose is 33 mg/kg at a true body weight of 9 kg. The maximal dose of 60 mg/kg can be seen at both 5 and 10 kg true body weight. The dosage now really lies within the proposed dose interval. The scale approach will be more precise than the current dose-pole approach provided a reasonably correct scale is used.

Doses according to the weight categorization in Fig. 7 are shown in Table 5. The weight intervals are chosen to minimize error in the dose, given that tablet halves are the smallest practical division of the praziquantel pill. In Fig. 7, this categorization of weight is used for both measurement series.

**Table 5:** Theoretically determined doses according to weight using tablet halves as the standard minimum dose fraction based on the weight of the child and with a dose never below 40 mg/kg

Weight (kg)	Number of tablets
12–18	1
19–26	1.5
27–33	2
34–41	2.5
42–48	3
49–56	3.5
57–63	4
64–71	4.5
72–79	5
80–87	5.5

If a given dose, using tablet halves should never fall below 40 mg/kg, this could be accomplished by a simple calibration of the scale in Table 5, as shown below in Table 6. Slightly increasing the dose means adjusting the weight thresholds, but still, the rather large weight intervals will remain. Now the maximum dose will slightly increase, but as can be seen in Table 6, the maximum is still within limits and fairly stable.

**Table 6:** Theoretically determined doses based on the weight of the child using tablet halves as the standard minimum tablet fraction and resulting in doses that are never below 40 mg/kg

Weight (kg)	Number of tablets	Max. dose (mg/kg)
12–15	1	50.0
16–22	1.5	56.3
23–30	2	52.2
31–37	2.5	48.4
38–45	3	47.4
46–52	3.5	45.6
53–60	4	45.3
61–67	4.5	44.3
68–75	5	44.1
76–82	5.5	43.4
83–90	6	43.4

## Discussion

We are advocating precision in praziquantel dosage because there is poor knowledge of praziquantel's side effects in mass treatment of *S. haematobium* contexts and also due to the possible risk for resistance development (9, 10). There is a large age-span in the group of children eligible for mass treatment with praziquantel. Our population consisted of schoolchildren 5–18 years old. In endemic countries, it might be difficult to identify the number of eligible children. The estimated proportion of the population in Tanzania between 5 and 15 years of age is 30%, which means that a little less than 14 million children are eligible for mass treatment of praziquantel (14).

If we focus on the difference expressed as mg/kg and based on our findings only select the number of children which would have been given doses with deviations from the target of more than 15 mg/kg, then approximately 3.7% [2.8–4.8], of the children would have been given >15 or <15 mg by the dose-pole compared to the weight approach. Assuming that our findings are representative for Tanzania, and that half of the children between 5 and 15 years of age would receive praziquantel in a mass treatment campaign, approximately 200,000–330,000 children would have received a dose dissimilar from the ‘gold standard’. The largest deviations from the recommended dose occur on the higher side of the dose scale.

According to Hall et al. (4), the dose-pole would be good enough for a majority of schoolchildren, but the association between weight and height could get more complicated in endemic child populations where you might find malnutrition (15). There are, however, possibilities for improving the dose-pole. For some child populations, a mass treatment strategy, where extra concessions are made for those children who are lean or heavy for their height, might be preferable.

Follow-up studies concerning optimal dosage, side effects and effectiveness of praziquantel in mass treatment programs using the dose-pole seem to be non-existent. According to WHO, the dose-pole delivers at least 40 mg/kg (1), which is *not* correct. What is needed to efficiently eradicate or at least reduce the number of parasites for different categories of individuals and yet minimize the possibility for the development of resistance, is not clear. Montresor (16) argues that the cure rate is not valid as an indicator for assessing drug efficacy and impact of preventive chemotherapy against schistosomiasis. This seems reasonable if the possibility of resistance development is not associated with the dosage.

There have been proposals to include even smaller children than currently recommended in mass treatment programs (13, 17). The idea is to include children from 60 cm of height in two new height intervals at the lower end of the dose-pole. As seen in our findings, it is exactly in this range of the height scale that the largest risk for deviations in dosage exists. The proposed extended dose-pole is not designed to take this into account; the suggestion is to distribute ¾ of a tablet to children between 83 or 84 and 99 cm, thereby introducing the need to use segments smaller than half a tablet.

The current dose-pole does not provide doses with higher precision than half a tablet. When adopting this half tablet strategy in a weight approach, the scale used does not need to be precise to the kilogram. The scale only has to correctly identify every 6th kg, which should make the weighing process rather simple. Given a properly functioning scale, the more extreme misclassifications would then be greatly reduced. From a practical point of view, weighing scales have already been constructed to show the number of tablets instead of kg in analogy to the dose-pole. Such modified scales have been proved to be robust in field tests (18). The conclusion is that the requirements for such a weighing device are not as high as previously assumed.

If one accepts the deviations we have observed using the dose-pole, not seeing the other alternatives, one also has to acknowledge the potential risks with the method. Our results, however, show that there indeed exists an alternative which gives higher precision.

## Conclusions and interpretation

The WHO dose-pole based on height for praziquantel dosing will deliver different doses for a given weight of a child since for each weight different lengths of children will always be a fact. Our study shows that children with the same weight could qualify for up to four different dose levels of praziquantel. The largest variation of doses based on the WHO dose-pole will be found in children below 20 kg of bodyweight. Using bodyweight and tablet halves as the smallest tablet division unit to determine the doses of praziquantel, one only has to identify every 6th kilogram of bodyweight; the doses will then vary a lot less than when using the WHO dose-pole.
